# Nature as a living presence: Drawings by Tupinambá and New York Children

**DOI:** 10.1371/journal.pone.0203870

**Published:** 2018-10-10

**Authors:** Christiana Profice

**Affiliations:** Department of Philosophy and Human Sciences, Universidade Estadual de Santa Cruz, BA, Ilhéus, Brazil; Kyoto University, JAPAN

## Abstract

Child–nature interaction has undergone drastic changes in modern history, from a free outdoor childhood to a confined daily life connected to electronic devices, with negative consequences to development and well-being. Any resulting lack of connection to the natural environment can hamper involvement in solving environmental problems. This research attempted to assess children’s perceptions of nature, as well as their feelings and values. Six- to 14-year-old children from the Tupinambá group (n = 91), an indigenous society in Brazil, and from New York (n = 54) drew pictures of nature and answered five questions about their drawings, feelings, and values in regard to natural environments. Quantitative (descriptive) and qualitative (content) analyses of the drawings were carried out, and their liveness and animism were estimated. The answers given by children to the questions about nature were organized into emerging categories from the data. The Tupinambá children’s drawings were generally livelier than those of the New York children. However, the difference failed to reach statistical significance among the younger children, and the difference only approached significance among the older children. The drawings of the Tupinambá contained more animism, depicting non-humans and non-animals with facial expressions, than those of the New Yorkers. Compared with the New Yorkers, the Tupinambá children more often included human constructions such as roads and houses in their drawings. The indigenous children more often saw human and non-human elements as integrated compared with the nonindigenous children. The study reinforces theoretical tendencies about the environmental perception of children in relation to the natural environment and highlights peculiarities of the participating groups, indicating relevant questions for future investigations.

## Introduction

Anthropologists and social thinkers generally believe that the human experience of nature has undergone radical changes over our species’ evolutionary history [1–3]. Until the creation of agriculture and the domestication of animals, humans felt a close connection to the life around them. Children, relatively few in number, participated with their families and small groups in food gathering and hunting, freely roaming the lands. With the advent of agriculture and the domestication of non-human animals, humans began to perceive themselves as separate from the rest of nature, which they could control and subjugate. The agricultural and pastoral communities then adopted a less nomadic lifestyle, and children began to form numerous groups, collaborating in both field activities and domestic functions.

However, children continued to spend most of their days outdoors. Until the past two centuries, as a consequence of urbanization and industrialization, significant numbers of children came to live most of their lives indoors (inside houses, apartments, and schools). Until the middle of the 20th century, pioneering thinkers like Rachel Carson and Maria Montessori expressed alarm that modern children were being imprisoned in indoor, artificial environments. These writers argued that children feel a natural connection to nature [4–6]. To children, nature is a living presence that invites energetic exploration and contemplation. If children are isolated from nature, as is the case in modern life, then their emotional and spiritual development suffers [7].

The other side of this issue concerns the degradation of natural environments, which is the most serious consequence of human urban lifestyles and affects all beings on Earth. Environmentalists and environment–person researchers are concerned about the children–nature connection. In a nutshell, children need to reconnect with nature so they can perceive environmental problems and become involved in their solutions [8]. The adoption of an urbanized lifestyle goes in the opposite direction of biophilia, namely, humans are attached to the living world and generate phenomena such as videophilia and technophilia, which both refer to the prevalence of everyday activities associated with electronic devices that are usually connected to the Internet [9–11].

With respect to biophilia, many controversies about whether this trend is innate or not nurture animate polemics between naturalists and culturalists. For the purposes of our present study, we recognize biophilia as a concept in which human beings are attracted to the living world, and interactions with other natural beings are fundamental for us to become humans; finally, we share elementary emotions (such as fear and security) with animals [4,12].

In this work, data from a research conducted with children are discussed to approach their perceptions of nature, as well as their knowledge and feelings about natural beings and environments. Through drawings and interviews of two groups, one composed of Tupinambá indigenous children (Brazil) and another of children living in New York (USA), we may determine their view of the natural world and their feelings.

### Children drawing nature

Before presenting our own research, we must highlight that drawings are widely employed as a research strategy to assess children’s environmental perception of nature [13–16]. In general, drawings possess validity in the sense that children’s accurate portrayal of settings is associated with time spent in such settings [17–21]. Many researchers suggest that children’s drawings can express the value they place on nature [22]. Regarding environmental issues, children should actively participate in setting priorities and making decisions. In this sense, drawings are widely used as a strategy to assess children’s perceptions and values in relation to their environment to facilitate the understanding of the child’s perspective and its participation in solving environmental problems [23, 24].

Starting from a phenomenological approach, a Swedish researcher asked 109 children between seven and 16 years old to draw what came to mind when hearing the word “environment.” Approximately 50% of all the drawings were placed in the category labeled as “the theme good world,” with prevalence of the youngest and the girls, adopting either a biocentric or an anthropocentric perspective [22]. In a research conducted on Brazilian children living in the Atlantic Forest by the author of this paper, 209 drawings and interviews were analyzed [17]. Each child drew nearly 13 elements; those under 9 years drew an average of 11.79, and those over 9 years in age drew an average of 14.21. Among the mentioned animal and vegetal species, 8% were domestic, whereas 92% were wild. Regarding the reported plants, 89% were generally referred to as tree, flower, leaf, or bush, and 11% had their species distinguished. People were present only in 25.8% of the drawings. The vast majority of children had chosen not to include humans in their landscapes. This study revealed that the participants know the landscape where they live, the beings living there, and local environmental issues [17]. Another study analyzed 9- to 11-year-old children’s drawings of tropical rainforests immediately before and after a visit to the Humid Tropics Biome at the Eden Project, Cornwall, UK. In the pre-visit drawings, animals were more present while plants were rare. After the visit to the tropical rainforest of the botanical garden, the animals became rare while the plants were more frequent. This study reinforces the idea that children’s drawings can be an effective method of assessing some aspects of their learning [20]. In the same direction, studies with children’s drawings demonstrate that exposure to the outdoors in green environments considerably enriches their interest and knowledge about plants [21].

Our investigation aimed to understand how children from different backgrounds–Tupinambá (Brazil) and New York (EUA)—perceive nature through the appreciation of their feelings and their knowledge about natural environments and beings.

## Methods

### Participants

Two samples of children, both ranging in age from 6 years to 14 years, participated in this study. The first sample consisted of 91 Tupinambá indigenous children. Drawing sessions and interviews were conducted in 10 indigenous schools between 2013 and 2016. Our initial contact with the Tupinambá took place in 2010. Since then, we have obtained formal authorization from the indigenous leadership to ask children to draw pictures, conduct interviews with them, and observe their activities in schools and in their community. The Tupinambá, who have had contact with European settlers since 1500, follow the modern practice of sending their children to schools taught in Portuguese, but the schools seek to maintain indigenous customs such as body art and circular dances. The Tupinambá also go barefoot and, according to ancient custom, the entire community gives children names associated with animals and plants. Economically, the Tupinambá continue to engage in collective cassava flour production and use plants for medicine. The Tupinambá also spend long stretches of time simply observing nature, which is a practice that seems old.

The second sample consisted of 53 children in New York. Drawings and interviews were conducted in 2016. All but four attended a private Catholic school in the New York City Borough of Queens. The school is located in a neighborhood known for its ethnic diversity. Our study distinguished 30 different nationalities cited by children as their background. Unlike research conducted with indigenous communities in which we visited 10 schools for almost 3 years, data collection among New York children was much narrower in terms of time and scope in the academic context of a postdoctoral stage. Although the enormous differences between the samples is recognized, a first comparative approximation can be useful for the understanding of the cultural differences with respect to children’s environmental perception of nature.

The two samples were similar in gender: 49.5% of the Tupinambá sample and 47.2% of the New York sample were girls (Table 1). Matching the samples by age became problematic. In the course of the New York school interviews, a winter storm disrupted school schedules, and some children could not be interviewed to match the Tupinambá sample by age. Therefore, four more children were interviewed in Manhattan and upstate New York to achieve a good balance between samples.

**Table 1:** Participant’s frequencies by country and gender.

	Girls		Boys		Total	
	n	%	n	%	N	%
BRAZIL	45	49.5	46	50.5	91	100
USA	25	47.2	28	52.8	53	100
**Total**	**70**	**48,6**	**74**	**51,4**	**144**	**100**

Despite these efforts, the Tupinambá sample was slightly younger than the New York sample. The mean age of the Tupinambá sample was 8.55 (SD = 1.86) compared with 10.40 (SD = 2.47) for the New York sample. For this reason and following previous studies with similar methodology, the samples were split into age group. The first group comprised children between 6 and 9 years old, and the second group consisted of children aged between 11 and 14 years (Table 2). As a consequence, any significant Tupinambá/New York differences can, theoretically, reflect sample differences in age. The efforts to evaluate this possibility are reported in the Results section (beginning with the variable labeled “Liveliness”).

**Table 2:** Participant’s frequencies by country and age range.

	(6–9)		(10–14)		Total	
	n	%	n	%	N	%
BRAZIL	63	69.2	28	30.8	91	100
USA	15	28.3	38	71.7	53	100
**Total**	**78**	**54.1**	**66**	**45.9**	**144**	**100**

### Ethics statement

All children were allowed to participate in the survey both from their parents or guardians and from the school authority who formally granted this permission by signing an informed consent term. For the study with the Tupinambá children, we obtained authorization from the Committee of Ethics in Research with Human Beings / Brazil—CAAE: 17984513.6.0000.5526.

### Procedures

As stated above, drawing sessions and interviews with Tupinambá children were carried out in the context of broad research on indigenous childhood. The author and her team of five undergraduate students and a Master’s student visited the participating schools from 2013 to 2016. Compared with the children of New York, the Brazilian Tupinambá group answered to a much larger questionnaire about their knowledge of biodiversity and questions regarding their school and indigenous condition. For the New York sample, the author and two academically advanced high school students held the drawing sessions in the school library. All children drew in small groups that varied from 6 children to 18 children. The additional four children living in Manhattan or upstate New York were asked to draw pictures individually by a faculty colleague.

No predetermined time limit was given for the children to draw. The indigenous schools, due to their own dynamics, did not set strict timetables for the duration of research activities. As the children finished their drawings, they were interviewed by one of the team members, usually made up of five people, which meant that the waiting time between drawing and interview was as short as possible. Notably, each child's drawing time was different, which made it possible, with rare exceptions, to always have an available interviewer. At the New York school, the only time available was while the class stayed in the library, which was 40 min. In this way, these interviews were objective and punctual and conducted by a team of only two interviewers. American children who were interviewed out of the school had no time restrictions. Without the systematic recording of time, one can estimate, from empirical experience, that the time a child takes to draw the suggested theme is between 10 and 15 min, although enormous individual variations may exist. This aspect should be observed in future investigations.

The variable of Drawn Elements was subjected to normality test (Kolmogorof–Smirnov) in the Tupinambá and New York groups separately (p = 0.00 and 0.02, respectively), indicating that the variable did not follow a regular distribution. Therefore, nonparametric tests (Mann–Whitney U tests and chi-square tests) were performed to estimate statistical significance according to social group (Tupinambá vs. New York), gender, and age group.

Every child was given a box and pencils with 12 colors and a sheet of 21 cm × 21 cm sheet of paper. The children were asked to draw a picture of nature and of nature around them. They were instructed to draw whatever they wanted and concentrate on their own drawings, regardless of the artwork of their friends and classmates. The researchers emphasized that all children can draw, and nothing in the drawings or answers was right or wrong.

After the children finished their drawings, we interviewed all individuals using to the following script. First, each child was asked his or her name, age, background, and whether the task is easy or difficult. Then, the child was asked to tell us about his or her drawings. The following five questions were asked to explore the children’s feelings about nature: What’s your feeling about nature? What’s the usefulness of nature? What’s good in nature? What’s bad in nature? What should the relationship between humans and nature be like? Finally, each child was asked if he or she had anything to add and if we could keep her or his drawing.

### Data analyses

Drawings were analyzed from quantitative and qualitative categories. To assess the amount of detail in the drawings, the total number of each element (e.g., each animal, each human, the sky, the sun, and the rain) was counted. These quantitative categories separately indicate the frequency of human, plant, animal, and celestial elements in the pictures. Elements of the natural landscape (such as mountains and rivers) and those that refer to construction, roads, and artificial objects were also noted. Together, these elements make up the total elements drawn by each child. These quantitative categories were the same ones adopted in previous studies and reflect the totality and proportion of the elements drawn by each child [25].

Qualitative categories in the drawings indicate the amount of Liveliness and Animism. Given that Liveliness struck us as possibly involving a degree of subjectivity, we assessed *interrater reliability* between independent three raters with respect to 20 randomly selected participants for these variables. The raters knew the purpose of the study, but the drawings were analyzed with concealment of the country, age, or gender of the participants. Liveness was coded as None, Some, or A Lot. Liveliness demonstrates whether the drawing evokes the perception that its elements are actively interconnected (not just put together) and suggest that something is happening in the picture. Agreement among the three raters, indicated by Cohen’s Kappa, was 0.767. By contrast, Animism, scored as the presence of facial features in non-human and non-animal figures, gave little chance of subjective assessment. The three raters were in complete agreement with respect to the coding of this variable.

Answers given by the children to the five questions described above were divided into categories. These categories were not defined a priori and were organized from the answers given by the participants and will be discussed further during data analysis.

## Results

The research activities were carried out in different periods. The Tupinambá children participated in the research activities between the years of 2013 and 2016 when the 10 schools were visited at least twice each. The group of children in New York participated in the research only in the year 2016. In terms of methodological design this small temporal difference is not ideal but we believe that it is compensated by the high degree of interest that the comparison between the groups allowed us.

To assess age differences, the total sample was divided into younger children (ages 6–9) and older children (ages 10–14) following the trend observed in previous studies. The division into these two age groups most evenly divided the total sample. The older children drew more elements (mean = 14.62, SD = 9.22 vs. mean = 11.24, SD = 7.98). In addition, girls drew more elements than boys (mean = 14.50, SD = 9.66 vs. mean = 11.18, SD = 7.40). Mann–Whitney U-tests indicated that the age difference approached statistical significance (p < 0.06) and the gender difference was significant (p < 0.05). However, the number of elements in the Tupinambá and New York drawings (mean = 13.13, SD = 9.75 vs. mean = 12.21, SD = 6.58) did not significantly differ (p < 0.88). As previously indicated, the quantitative categories refer to the frequency of the following elements: people, plants, animals, celestial elements (e.g., sun and clouds), natural landscape (e.g., mountain and river), and human-built objects (e.g., houses and cars). The first part of the interview consisted of a description of the elements present in the drawing by the participating child. Each element was nominated by the child, which left very little room for ambiguity in its categorization. Simultaneously, the Tupinambá drawings, considered a group, revealed a highly differentiated conception of the biotic world. When talking about their drawings, the Tupinambá children cited a total of 30 different plant species compared with 12 cited by the New York children **(**Table 3). Similarly, the Tupinambá children referred to 43 different animal species compared with 22 described by the New York children.

**Table 3:** Biodiversity of drawings.

T Sample: Plants	NY Sample: Plants	T Sample: Animals	NY Sample: Animals
Leaves, Mango, Iamb, Flower, Blackberry, Rose, Coco palm, Grass, Carnivorous, Orange, Sunflower, Apple, Banana, Coco, Sugar cane, Avocado, Cashew, Jackfruit, Strawberry, Acerola, Pineapple, Cocoa, Melon, Grapefruit, Almond, Tree, Jabuticaba, Guava, Biriba, Jamelão.	Flower, Tree, Flower twig, Bush, Vine, Apple, Grass, Nest, Pumpkin, Berry tree, Leaves, Trunk.	Cat, Cow, Armadillo, Trace bird, Little bird, Generic fish, Jabuti, Turtle, Butterfly, Giraffe, Lion, Shark, Parakeet, Dog, Rufous, Hummingbird, Bee, Snake, Monkey, Alligator, Frog, Vulture, Jaguar, Parrot, Donkey, Deer, Horse, Chicken, Elephant, Catitu, Anteater, Paca, Ant, Wasp, Hawk, Bentevi, Mico, Sangue de boi, Penguin, Sloth, Raccoon, Capybara, Toucan.	Deer, Monkey, Dog, Horse, Wolf, Cat,Rabbit, Panda, Fox, Giraffe, Bear, Squirrel, Bird, Birdtrace, Egg, Owl, Woodpecker, Butterfly, Dinosaur, Fish, Earthworm, Ant.
**Total: 30**	**Total: 12**	**Total: 43**	**Total: 22**

### Human presence

We were interested in the extent to which children saw themselves or people as integrated into nature, rather than perceiving nature as a separate entity. Therefore, we counted the number of humans and human-built structures, such as buildings and roads, present in the nature drawings. Mann–Whitney U tests did not reveal significant age differences (under 9 years and 9 years and above) with respect to the number of humans (mean = 0.42, SD = 1.01 vs. mean = 61, SD = 2.43, p < 0.67) or human-built structures in the landscape (mean = 1.23, SD = 1.59 vs. mean = 0.94, SD = 1.38, p < 0.10). Similarly, no significant gender differences were found for these variables (mean = 0.30, SD = 0.74 vs. mean = 0.70, SD = 2.4, p < 0.74 for number of humans and mean = 1.14, SD = 1.05 vs. mean = 0.21, SD = 1.01, p < 0.21 for human-built structures). Humans were more frequently present in the drawings of the Tupinambá children than in the drawings of the New York children, but this difference was not statistically significant. However, the Tupinambá children included more human-built structures (mean = 1.38, SD = 1.53 vs. mean = 0.60, SD = 1.32, p < 0.01) than the New York children.

**Fig 1:**
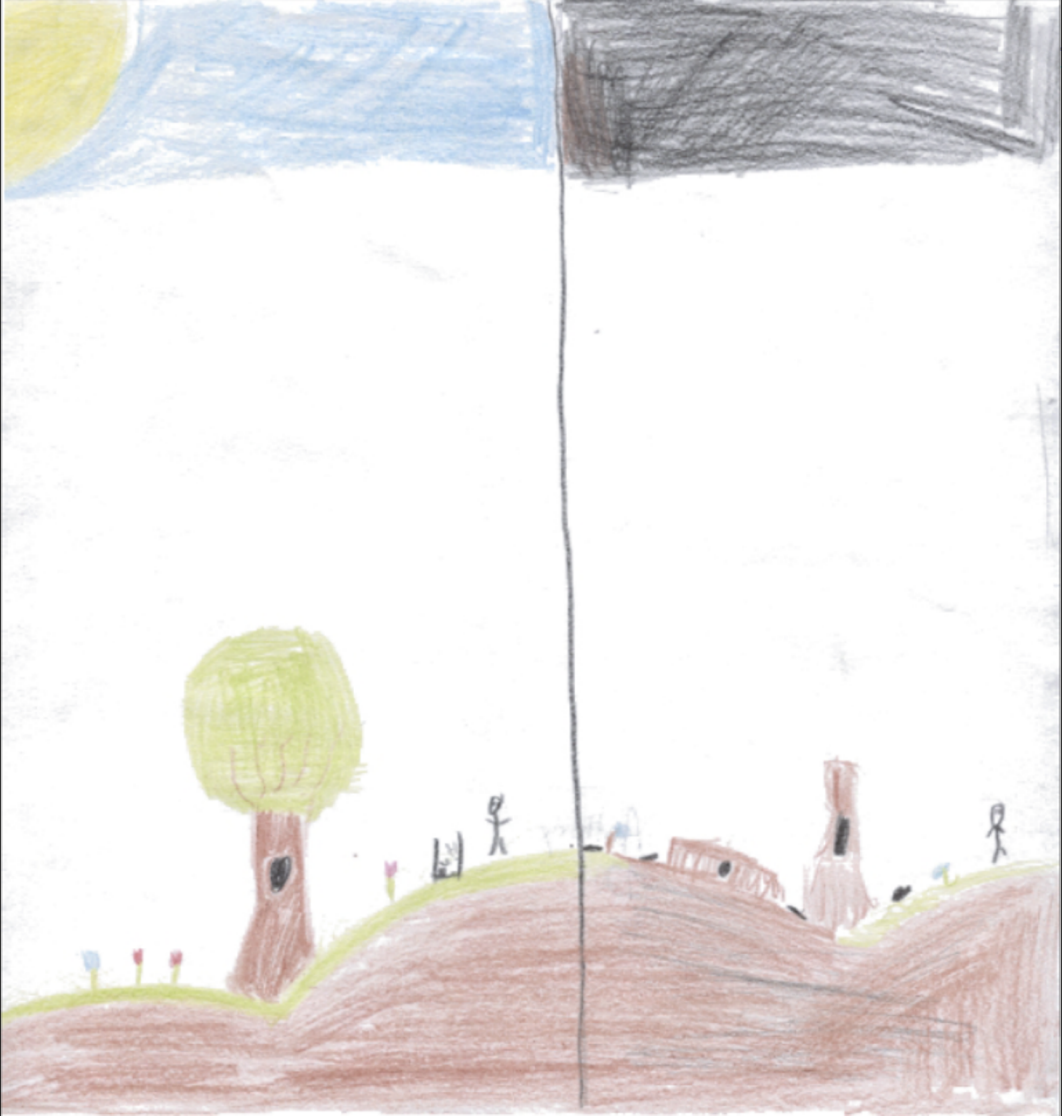
Nature. Boy, 12, New York.

To several New York children, nature was not just a land apart, but a land that humans have violated. For example, in the drawing below (Fig 1), a 12-year-old New York boy divided the sheet into “before” and “after.” On the left, we see a person in nature on a sunny day and blue sky. On the right, we see the effects of human intervention. Under a black sky, the tree is dead and only a few flowers have survived.

**Fig 2:**
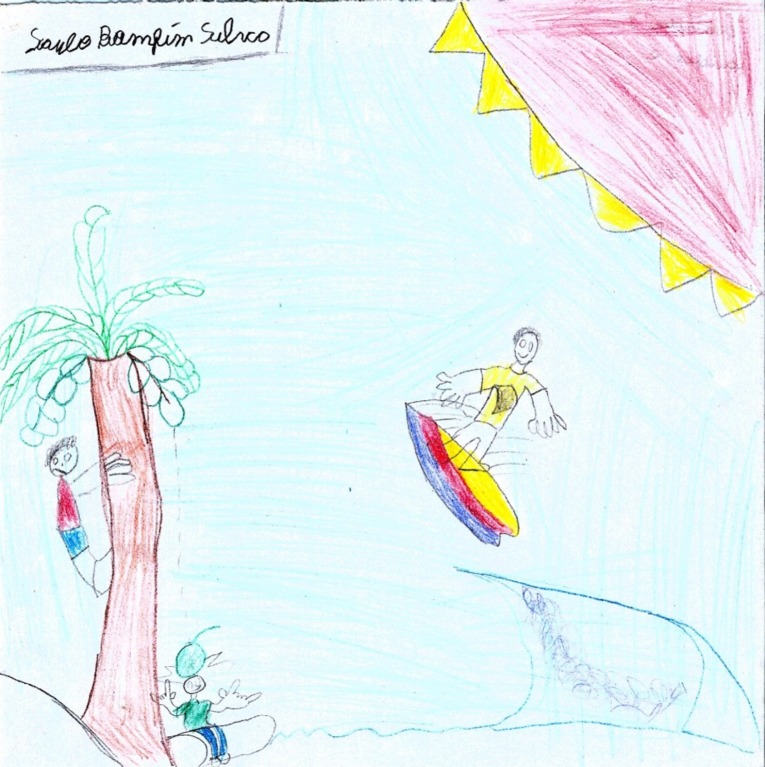
Nature. Boy, 11, Tupinambá.

By contrast, a vivid drawing by an 11-year-old Tupinambá boy shows people having fun and enjoying nature (Fig 2). One of the characters is surfing while the others are playing at a coconut tree. To this Tupinambá child, nature is simply a good place in which to live.

### Liveliness

**Fig 3:**
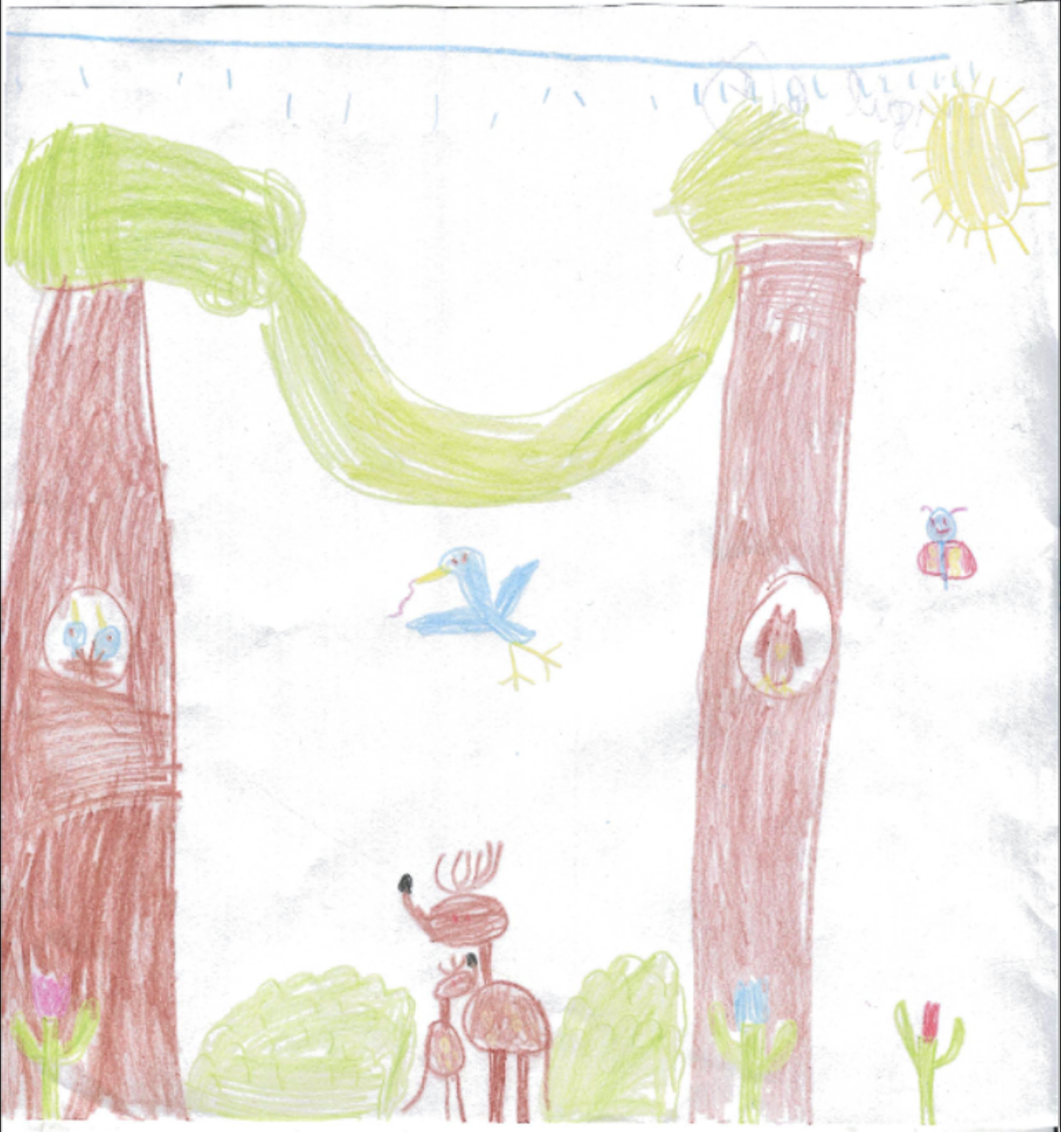
Nature. Girl, 6, New York.

One major concern was the extent to which the children might perceive nature as a living presence. Therefore, the drawings were evaluated in terms of their liveliness: “a lot,” “some,” or “none.” For example, the drawing below depicts “a lot” of liveness (Fig 3). A doe and fawn are snuggling, and a bird is carrying a worm. There is also rain at the top. Other drawings expressed just a little action, coded as “some” liveliness, or none at all.

The variable Liveness was significantly associated with age; the younger children’s drawings were livelier, as assessed by the chi-square test (p < 0.05). No gender difference was found with this variable.

Chi-square test also indicated that the Tupinambá children expressed significantly more liveliness (p < 0.01) than their New York counterparts. Given that the *younger* children’s drawings were significantly livelier, the Tupinambá**/**New York difference might have reflected the fact that the Tupinambá sample was younger. To evaluate this possibility, we compared Tupinambá versus New York drawings separately for the younger (9 and under) and older (10 and older) children. Chi-square tests indicated that Tupinambá/New York differences failed to reach statistical significance for the younger children but only approached significance for the older children (p < 0.06).

### Animism

Young children sometimes draw faces in the sun, plants, or elements such as clouds. Such drawings, which are widely considered to be examples of youthful animism, provided us with another indication of the extent to which the children perceived nature as full of life and emotion.

**Fig 4:**
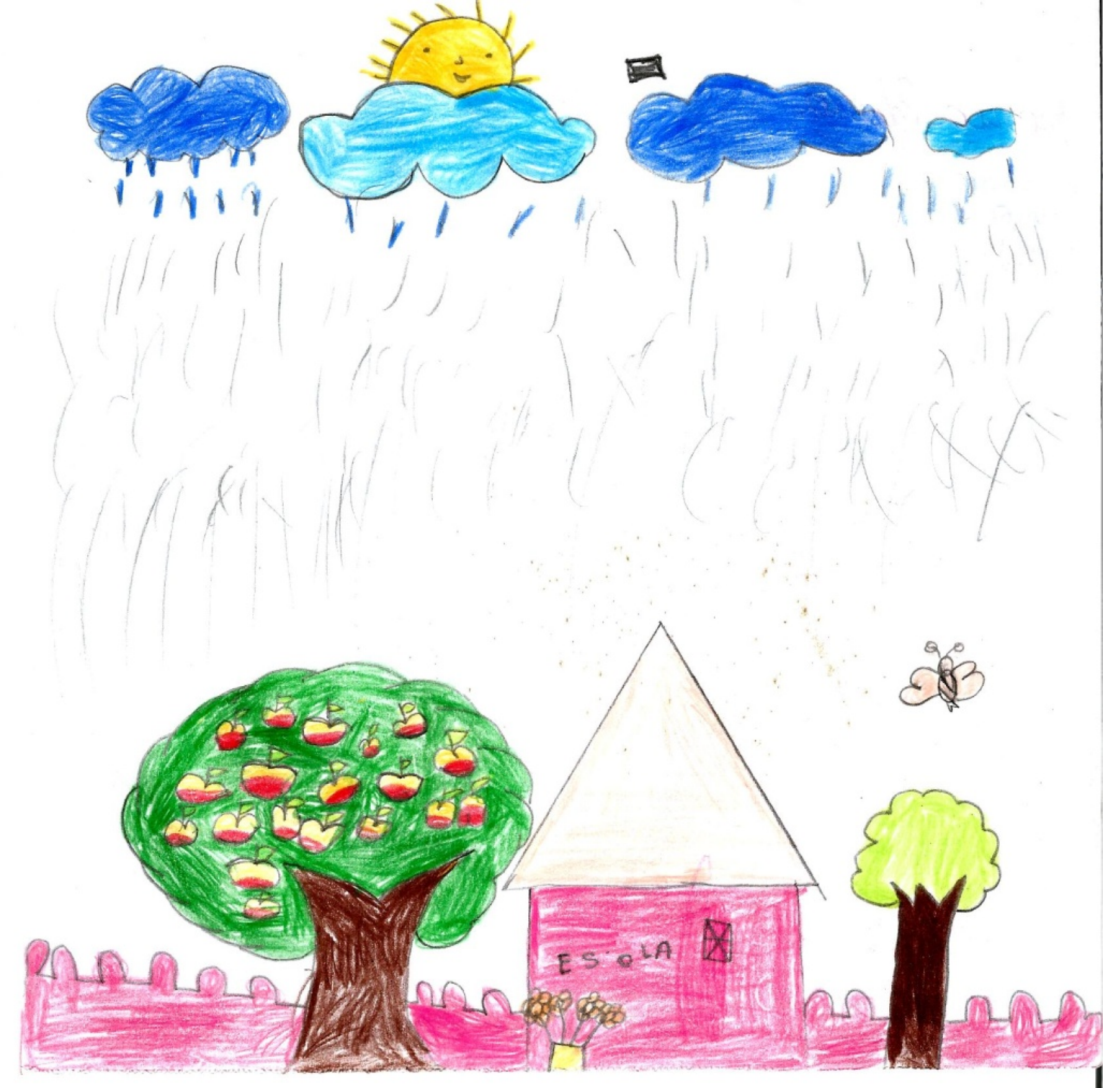
Nature. Girl, 8, Tupinambá.

Fig 4 illustrates animism. In this drawing by an 8-year-old Tupinambá girl, the sun is smiling (Fig 4). Notice also that a human structure, a school, appears in the drawing, illustrating a finding described earlier.

No significant difference in animism was found by gender, but animism was related to age: 74.4% of the children in the 9 and under age group versus 24.6% of those in the 10 and over age group drew a face in some non-human or non-animal being. This difference was significant at the 0.05 level as indicated by the chi-square test.

Even more striking was the difference between the Tupinambá and New York samples. Forty-one Tupinambá drawings (45.1% of the sample) but only two New York drawings (3.8% of the sample) contained animism (p < 0.01, chi-square test).

Tupinambá/New York differences also were significant for each age group examined separately. Among the younger children, 49% of the Tupinambá drawings contained animism, whereas only 7% of the New York children’s drawings contained animism. Among the older children, the Tupinambá/New York difference was 36% to 3%.

### Interview questions

As indicated earlier, the answers given to the five questions were grouped from the emerging content itself. For the first question, What is your feeling about nature?, responses were grouped into five categories. The first category refers to the answers that indicate good feelings such as happy, joy, beautiful, nice, good, wonderful, fun, peace, calm, nice place, and interesting and were organized around the label “Good feelings” (74.3%). The second category was called “Bad feelings” and encompasses terms such as fear, pain, and danger (1.4%). The third category indicates the answers that cited nature as a source of “Resources,” such as humans, animals, plants, and wind (2.8%). A fourth category nominated as “Ethical and Environmental concern” groups the answers that bring the idea of care, respect, and protection (12.5%). Finally, the fifth category labeled as “Other” brings together the children who did not respond and also the answers that did not fit into the previous categories (9%). Chi-square tests were performed, but no difference was considered significant between countries (p < 0.74), gender (p < 0.87), or age group (p < 0.15).

For the second question, What is the usefulness of nature?, four categories of answers were discerned. The first category, labeled “Resources and Ecosystem Services,” refers to food, shelter, air, water, and other products extracted from nature for human and animal use (49.3%). The second category, designated as “Human well-being,” refers to the use of nature to relax, calm, live, life, socialize, play, have fun, good feelings, and explore (25%). It differs from the previous category by not indicating a specific natural resource to be exploited. The third category, named “Environmental Problems,” indicates nature as an object of human concern and care but also its own ability to mitigate the environmental problems generated by humans (6.9%). The fourth category covers children who did not respond and answers that did not fit the options described above (18.8%).

The second interview questions revealed no significant differences between countries (p < 0.07), gender (p < 0.86), or age group (p < 0.06), as indicated by the chi-square test. Notably, a large portion of Tupinambá children (26.4%) did not answer or stated they “didn’t know,” suggesting that they did not understand the question. In the sample of New York children, this number represented only 5.7% of respondents.

The third question, What is good in nature?, provided four categories of responses. The first one was labeled “Animals, Plants, and Landscape Elements” and indicates the existence of these beings and natural environments without the mention of its usefulness for humans (49.3%). The second category, called “Resources, Ecosystem Services, and Human Well-being,” brings together responses that cite beings, environments, and natural resources that are expressly linked to human well-being (33.3%). The third category brings together global answers like “Everything (9%),” and the fourth category includes children who did not respond to this question and the answers that could not be classified in the previous categories like “I don’t know” (8.3%). Here a statistically significant difference (p < 0.00) between the Tupinambá and New York children was found in the overall distribution. More specifically, most Tupinambá children referred to natural beings and environments as good (56%) without any mention of usefulness to humans, whereas the New Yorkers most frequently pointed to the human benefits of natural resources (47.2%). The New York children were more utilitarian than their Tupinambá counterparts.

The fourth question, What is bad in nature?, suggested four categories of responses. The first category brings together “Aggressive Animals, Poison, Dangerous Plants, and Natural Disasters” (44.4%). A second category refers to human behaviors that damage nature (16%). The third category consists of those who answered “Nothing” (29.2%), and the fourth category indicates the children who did not respond or whose answers did not fit into the previous categories like “I don’t know” (10.4%). For this question, significant differences were noted between countries (p < 0.00), gender (p < 0.02), and age group (p < 0.001). With respect to country, most Tupinambá children stated that they found nothing bad in nature, whereas most Americans indicated that animals, plants, and natural disasters were bad. At the same time, 34% of American children said that what is bad in nature was the work of human beings, whereas that response was only given by 5.5% of the Tupinambá children. Nature for New York children was a much more dangerous and destroyed place than that for Tupinambá children. As mentioned with respect to the drawings, the New Yorkers, living in cities, imagined nature as an environment inhabited by menacing beings and people vulnerable to environmental disasters. Tupinambá children, living in nature, displayed a more benign view than the New Yorkers.

For the last question, How do you think the relationship between people and nature should be?, responses were classified as “Anthropocentric” (privileges for humans) (1.4%), “Ecocentric” (proposing a balance between humans and nature; 40.3%), “Biocentric” (prevailing nature; 45.8%), and “Undefined/No answer” (12.5%). The answers as nice or good were also classified as “Ecocentric” because they convey the idea that it should be good for both parties. Only two children took a definite anthropocentric approach. Here, differences were significant between the two countries (p < 0.01) and age groups (p < 0.05) but not between genders (p < 0.85).

## Discussion

The quantitative analyses of elements in each drawing and the presentation of the results on biodiversity revealed that the landscape of the Tupinambá children is more diverse than that of the children of New York. The greater specificity on the part of the Tupinambá children was too extensive to simply reflect the larger Tupinambá sample size. This difference certainly relates to the quantity and quality of interactions between the children of the two different groups with nature. Tupinambá children live in the natural environment and depend directly on their livelihood and grow much of their food, whereas New York children live in a large urban center where interactions with nature are scant and punctual, their families draw their sustenance from activities thatr are rarely linked to other living beings and acquire their food more or less processed in supermarkets. Simultaneously, we can not fail to consider the characteristics of the biomes in which the participating children live. Tupinambá children live in the Atlantic Forest biome with a high biodiversity level, whereas American children in New York live in the temperate deciduous forest with low biodiversity.

Numerous anthropological observations have suggested that indigenous societies regard nature as more alive compared with Western societies [3, 4, 7]. When the sample’s drawings were coded for Liveliness, the Tupinambá drawings were livelier, but the difference was not statistically significant among younger children, and it only approached significance among older children. Our judgments for the Liveliness variable were possibly too global and imprecise. Interrater reliability, while adequate, was not as high as we would have liked. By contrast, coding for another indication of life and emotion—the presence of animism—was easy and without disagreement. Animism, operationally defined as facial expressions in the sun, flowers, and other non-human and non-animal beings, appeared far more frequently among the Tupinambá drawings in both age groups than in the American drawings.

The magnitude of this difference was surprising. We realized that New York children, as part of Western culture, are gradually socialized to give up animism. They come to view nature in a more impersonal manner than their Tupinambá counterparts. However, society has grown accustomed to seeing animism in young children’s drawings, before the effects of socialization take hold. We were unprepared for the nearly total absence of animism, even among our younger children, in our New York sample. Further research is needed to ascertain if our finding is representative of a more prevalent trend in children’s drawings, and if so, to determine the causes of it.

The extensive prevalence of animism in the Tupinambá drawings drawings was unsurprising. True, scholars frequently described indigenous peoples as perceiving the entire world animalistically [25]. However, we had questions in the back of our minds. Was this description of animism really valid, or was it something of a stereotype? Even if animism was prevalent in indigenous cultures when they were studied in the past, is it still pervasive in today’s increasingly Westernized world? If the Tupinambá are representative, the answer is “yes.” Animism appeared in drawings with considerable regularity. It was even present among over a third of the older children. The results for animism indicate that Tupinambá culture views all of nature as a living, feeling presence.

The drawings and speeches of the Tupinambá and New York children manifest the natural scene as it is perceived by them. In particular, they are effective instruments used to assess children’s environmental perception and evaluate their ecological knowledge. However, results from research with different design methodologies do not yet allow consistent comparative analyses.

One aspect that caught the attention of New York children was a realistic tone in the face of the environmental problems caused by humanity. Environmental problems such as industrial pollution, deforestation for urban occupation and logging for paper production were mentioned in the participants’ responses to questions asked by a team of two interviewers. This generation is already fully aware of the vulnerability of the natural environment they are receiving from previous generations; they know that it is a disfigured and distant nature whose resources are running out. This worried maturity that can be found among American children goes hand in hand with the full awareness that this lack of nature does not do well for their own well-being and development. Thus, the children know they need more nature in their daily lives. Children in New York seem especially concerned about the pollution of the air they breathe than those in Tupinambá.

The Tupinambá children, in turn, are fully aware of the importance of nature to their survival and of their community and seem less stressed and worried about wide environmental problems. Their concern is the quality of the water that is available in the rivers and streams of the indigenous territory. Given that these children spend most of their time outdoors, the beings and processes of nature can influence a realistic view of the interaction between people and nature and promote their well-being.

## Conclusion

This research highlights differences among age groups in the way they perceive nature as well as how to express it through drawings and questionnaire responses. Results support the theory that a young child has an initial affective approach to nature that is expanding as the cognitive dimension is integrated. Thus, environmental education actions for children up to 9 years should seek strategies that are more sensitive and perceptive than those with a more cognitive and knowledge approach that should be used with older children. Gender differences were not conclusive in the research and should be further evaluated in future studies.

The concept of biophilia continues to be an important theoretical reference for the study of the interaction between children and nature. It is independent of being inborn or learned, and it can only be effective in a socio-cultural context that encourages it in everyday life. Currently, children are spending an increasing amount of time indoors using electronic devices and are spending less time in outdoor environments in interaction with nature and its beings. Humans need the natural world to become humans; by contrast, we are creating new generations that do not feel like they belong in nature and already present disturbances in their physical and mental health. Through this research, the Tupinambá and New York children warn us that the interaction between people and nature is essential to address environmental problems and ensure healthy development and well-being.

As demonstrated in this research, the proximity to nature and its cultural references influence children’s drawings. Unfortunately, children living in a big city like New York do not bring liveness to nature in their drawings when we know the importance of nature for the development and well-being of all children. This kind of result should be better explored by future investigations. However, all children are aware of current environmental problems and are willing to participate in their solution. This opportunity is one that we should not waste. Engaging children in community decision making and environmental management is an effective way to make them greener and livelier.
